# Drug prioritization identifies panobinostat as a tailored treatment element for patients with metastatic hepatoblastoma

**DOI:** 10.1186/s13046-024-03221-6

**Published:** 2024-11-12

**Authors:** Salih Demir, Alina Hotes, Tanja Schmid, Stefano Cairo, Emilie Indersie, Claudio Pisano, Eiso Hiyama, Tomoro Hishiki, Christian Vokuhl, Sophie Branchereau, Penelope Brock, Irene Schmid, József Zsiros, Roland Kappler

**Affiliations:** 1 Department of Pediatric Surgery, Dr. von Hauner Children’s Hospital, University Hospital, LMU Munich, Lindwurmstreet 2a, Munich, 80337 Germany; 2 XenTech, Evry, France; 3 Champions Oncology, Inc, Rockville, MD USA; 4 Biogem, Institute of Molecular Biology and Genetics, Via Camporeale, Ariano Irpino, Italy; 5 Natural Science Center for Basic Research and Development, Hiroshima University, Hiroshima, Japan; 6 Department of Pediatric Surgery, Chiba University Graduate School of Medicine, Chiba, Japan; 7 Institute of Pathology, University Hospital Bonn, Bonn, Germany; 8 Bicêtre Hospital, AP-HP Paris Saclay University, Paris, France; 9 Department of Paediatric Oncology, Great Ormond Street Hospital for Children, NHS Foundation Trust, London, UK; 10 Department of Pediatrics, Dr. von Hauner Children’s Hospital, University Hospital, LMU Munich, Munich, Germany; 11 Princess Máxima Center for Pediatric Oncology, Utrecht, The Netherlands

**Keywords:** Hepatoblastoma, Metastasis, Panobinostat, Therapy, MYC

## Abstract

**Background:**

Patients with metastatic hepatoblastoma are treated with severely toxic first-line chemotherapies in combination with surgery. Yet, inadequate response of lung metastases to neo-adjuvant chemotherapy still compromises patient outcomes making new treatment strategies, tailored to more efficient lung clearance, mandatory.

**Methods:**

We harnessed a comprehensive patient-derived xenograft platform and a variety of in vitro and in vivo assays to establish the preclinical and biological rationale for a new drug for patients with metastatic hepatoblastoma.

**Results:**

The testing of a library of established drugs on patient-derived xenografts identified histone deacetylase inhibitors, most notably panobinostat, to be highly efficacious on hepatoblastoma cells, as compared to non-cancerous cells. Molecularly, the anti-tumor effect of panobinostat is mediated by posttranslational obstruction of the MYC oncoprotein as a result of dual specificity phosphatase 1 upregulation, thereby leading to growth inhibition and programmed cell death. Of clinical importance, upregulation of the MYC target gene nucleophosmin 1 is indicative of response to panobinostat and associated with metastatic disease in patients with hepatoblastoma. The combination of panobinostat with the current SIOPEL 4 induction protocol, consisting of cisplatin and doxorubicin, revealed high synergies already at low nanomolar levels. The simulation of a clinical trial, with this combination therapy, in patient-derived xenograft models, and ultimately heterotypic lung metastasis mimics clearly underscored the potency of this approach.

**Conclusion:**

Integrated studies define MYC inhibition by panobinostat as a novel treatment element to be introduced into the therapeutic strategy for patients with metastatic hepatoblastoma.

**Supplementary Information:**

The online version contains supplementary material available at 10.1186/s13046-024-03221-6.

## Background

Hepatoblastoma (HB) is the most common form of primary childhood liver cancer and is usually seen in young children and infants [1]. The combination of cisplatin monotherapy and surgery is the standard of care for children with standard-risk disease and results in good long-term survival [2]. However, the outcome of children with high-risk disease, including metastatic spread to the lung still remains unsatisfactory, although the prognosis of these patients has been increased by the introduction of dose dense cisplatin-based chemotherapy [3, 4]. The main reason for treatment failure is the inadequate response of the metastases to neo-adjuvant chemotherapy in a significant proportion of patients, which prevents complete surgical resection of all tumor foci and/or leads to refractory, progressive or recurrent disease. In addition, the high toxicity of chemotherapy leads to serious long-term sequelae in a large proportion of survivors, such as irreversible hearing loss, renal insufficiencies and heart failure, thereby impairing their quality of life [5].

The development of new therapeutic approaches with particular regard to metastatic disease has been hampered by the very limited number of chemotherapeutic drugs with proven efficacy in HB and the relatively high toxicity of the current treatment. Even worse, for patients who fail their treatment there are no sufficiently efficacious second line or salvage options available. Thus, the urge to establish new drugs for metastatic HB is immense. In consideration of the fact that the current Pediatric Hepatic International Tumor Trial (PHITT) will be completed at the end of 2026 [6], in which children with initial metastases were treated according to the previously established SIOPEL 4 regimen [3], a Childhood Liver Tumors Strategy Group (SIOPEL)-based working group was initiated in 2022 to design a new clinical trial, tentatively by using targeted therapeutic options or by repositioning medications already approved for other indications.

In this study, we made use of a newly developed comprehensive in vitro drug-testing platform to prioritize putative drugs, including histone deacetylase (HDAC) inhibitors, for their implementation into a new clinical trial for patients with metastatic HB. We furthermore determined the preclinical and molecular background for using the top-ranking drug panobinostat in combination with the SIOPEL 4 therapy.

## Methods

### Cell lines and PDX lines

We used the five established liver cancer cell lines HUH6, HUH7, HepG2, Hep3B, and HepT1 as well as seven patient-derived xenograft (PDX) cell lines, which have been generated by Xentech, Evry, France. Moreover, primary adult and neonatal human dermal fibroblasts and human bronchial epithelial cells were utilized as healthy controls. Cell lines were authenticated by Sanger sequencing of CTNNB1 mutations [7] and routinely tested negative for mycoplasma contamination. Detailed information on the cell lines and culturing conditions are reported in Suppl. Table 1.

### In vitro drug testing

5 × 10^4^ cells were seeded in 96-well plates and exposed to increasing drug concentrations ranging from 5 nM to 100 µM in 1:3 dilutions for 48h. MTT (3-(4, 5-dimethylthiazol-2-yl)-2, 5-diphenyltetrazolium bromide)-based viability assay was performed as described [8] and nonlinear regression of drug response curves were plotted in relation to DMSO control. Half-maximal inhibitory concentrations (IC50) and area under the curve (AUC) values were calculated using GraphPad Prism 8 (GraphPad Software, San Diego, CA). Z-scores were calculated using the Z = [(Individual AUC value)-(mean AUC)]/ standard deviation formula. Therapeutic window scores were calculated by using the TWS = (mean AUC of tumor lines)/ (mean AUC of healthy controls) formula. Detailed information on drugs can be found in Suppl. Table 2.

### Expression analysis

Association studies of RNA expression and patient information were conducted on the publicly available GSE131329 data set of the JPLT-2 trial [9], which was retrieved from the R2 genomics analysis and visualization platform (http://r2.amc.nl).

### Western blot analysis

Whole cell lysates and nuclear extracts were isolated as previously described [10]. 20 µg of proteins were denatured at 70°C for 10 min, separated in 4–20% gradient gel (Thermo Fisher, Waltham, MA) and transferred to nitrocellulose membranes (BioRad, Hercules, CA). Membranes were incubated overnight at 4°C with primary antibody dilutions (detailed information in Suppl. Table 3). Following 1h incubation with respective secondary antibodies, proteins were detected with enhanced chemiluminescence detection reagent (Amersham Biosciences, Amersham, UK) using the ChemiDoc XRS + imaging system (Bio-Rad).

### Apoptosis assay

Apoptosis was detected by CellEvent™ Caspase-3/7 Green Detection Reagent (Thermo Fisher) according to the manufacturer’s instructions by detecting cells that were positive for active substrates of caspase 3 and 7 using the EVOS M7000 imaging system (Thermo Fisher).

### Short- and long-term growth of tumor cells

Short-term growth was detected with Click-iT EdU cell proliferation kit (Thermo Fisher) according to the manufacturer’s instructions and microscopic images were obtained by using the EVOS M7000 imaging system (Thermo Fisher). Long-term growth of the tumor cells was detected by colony formation assay by seeding 2 × 10^3^ cells/well in 6-well plates, then exposing them to either DMSO or 1 nM panobinostat for 7 days. Upon formation of colonies, cells were stained with 0.5% crystal violet (Sigma-Aldrich, St. Louis, MO) in 20% methanol for 2h. Images of colonies were taken by GelJet Imager and EVOS M7000 (Thermo Fisher) for overview and magnified examination, respectively.

### Live/death staining of tumor spheroids

A total of 1 × 10^3^ cells in 100 µl/well were seeded into ultra-low attachment round-bottom 96-well plates (Corning, Corning, NY) for 5 days until spheroids were formed. Then, established spheroids were exposed to 1 nM panobinostat or DMSO and images were captured at day 4. Thereafter, live/death detection was performed by simultaneously staining the spheroids with 3 µM of the viability dye calcein-AM (BioLegend, San Diego, CA) and 2 μg/ml of the death dye propidium iodide (Sigma-Aldrich) at room temperature for 20 min. All microscopy images were taken with EVOS M7000 (Thermo Fisher).

### RNA sequencing analyses

RNA isolation, library preparation, HiSeq2500 sequencing (Illumina; San Diego, California, USA), read alignment and quantification was performed as described previously [11]. The Bioconductor (RRID:SCR_006442) package DESeq2 (RRID:SCR_000154) was applied for the normalization and the analysis of differential expressed genes between treated and untreated samples. Enriched pathways in hallmark gene sets of the human molecular signature database (MSigDB) were detected by gene set enrichment analysis (GSEA) (https://www.gsea-msidb.org). Differentially expressed genes > twofold with a *p*-value < 0.05 were used in the Enrichr database (RRID:SCR_001575) to identify enriched hub proteins (protein–protein-interaction hub) and to obtain clustergrams showing upregulated partner proteins (https://maayanlab.cloud/Enrichr/). Significantly upregulated genes were also used as input for the kinase enrichment analysis v3 (KEA3) database (https://maayanlab.cloud/kea3). The protein–protein interaction network was generated by using the STRING web tool v12, RRID:SCR_005223 (https://string-db.org).

### Oncogene-driven liver tumor mouse models

Liver-specific expression of the human *MYC* and the mutant mouse *Kras* oncogenes was achieved by crossing *LSL-MYC* [12] and *LSL-Kras*^*G12D*^ [13] transgenic mice with Cre^Alb^ mice [14]. The generation of liver tumor mouse models was approved by the Regierung von Oberbayern (ROB-55.2–2532.Vet_02-19–63). Cre^Alb^Myc and Cre^Alb^Kras mice spontaneously developed liver cancer at approximately 1 and 11 months old, respectively. Liver tumors were dissected from animals, immediately snap-frozen, and stored in liquid nitrogen for up to 12 months. Tumors were histologically diagnosed on hematoxylin (Roth) and eosin (Sigma-Aldrich) stained cryosections by a trained paidopathologist. For immunohistochemistry, cryosections with 5 µm-thickness were fixed with 4% paraformaldehyde for 15 min, permeabilized with 0.5% Triton-X-100 for 60 min and blocked in PBST containing 3% BSA and 0.1% glycine for 30 min at room temperature. Slides were then incubated with MYC, EPCAM and GPC3 antibodies overnight at 4°C and incubated with Alexa Fluor 647, 488 and 555 secondary antibodies for 1h at room temperature the following day, respectively (detailed information in Suppl. Table 3). All images were captured with EVOS M7000 (Thermo Fisher).

### Primary cell cultures

To generate primary cell cultures from oncogene-driven liver tumor mouse models, parts of the freshly dissected liver lesions from sacrificed animals were washed in PBS and immediately minced with a scalpel in a 6-well culture plate. Dissociated tumors were covered with a drop of advanced DMEM/F12 containing 10 mg/L insulin supplemented with 10% fetal bovine serum, 1% penicillin/streptomycin, 1% L-glutamine (all from Thermo Fisher), as well as 20 µM Y-27632 (Selleckchem, Chesterbrook, PA) overnight to prevent floating of tumor pieces in the media and allowing for attachment of cells to the culture plate surface. The next day, 2 ml of fresh media was supplied and cells incubated with media exchange every 3–4 days for approximately two weeks. Vital tumor cells were then detached by selective short-term trypsinization and further cultivated for several passages until used in treatment experiments.

### Heterotypic lung metastasis mimics

For characterization purposes, BEAS-2B, HDFn and HB cells were seeded into ultra-low attachment round-bottom 96-well plates for three days to form spheroids, then washed with PBS, and subsequently fixed in 4% paraformaldehyde for 60 min at room temperature, permeabilized with 0.5% Triton-X-100 overnight at 4°C, and blocked in PBST containing 10% BSA for 30 min at room temperature. Spheroids were then incubated with antibodies against surfactant protein C (SP-C) as pulmonary marker, vimentin (VIM) as mesenchymal marker, and alpha-feto protein (AFP) as HB marker overnight at 4°C (detailed information in Suppl. Table 3). Fluorescent signals were detected by incubating spheroids with respective Alexa Fluor 647-labeled secondary antibodies and counterstaining with Hoechst 33342 (Thermo Fisher) for 1h at room temperature. Images of three-dimensional spheroids were captured with the EVOS M7000 system (Thermo Fisher) and pseudo-colorized using ImageJ (https://imagej.nih.gov/ij/).

In order to distinguish different cell types in the two assembly methods used for lung mimic modeling, BEAS-2B, HDFn and HB cells were labeled with Vybrant DiO, Dil and DiD, respectively, according to the manufacturer’s instructions (Thermo Fisher). Briefly, 1 × 10^6^ cells were resuspended in 1 ml serum-free medium and incubated with 20 µl of dye solution in the dark for 30 min. For the mixed-spheroid-metastasis model, 1 × 10^3^ cells from each cell type labelled with individual tracking dyes were mixed in equal proportions and seeded into ultra-low attachment round-bottom 96-well plates for three days. Established spheres mimicking lung metastases were then exposed to either DMSO or 1 nM panobinostat for three days. For the merged-spheroid-metastasis model, 1 × 10^3^ cells from each individually labelled cell type were separately seeded into ultra-low attachment round-bottom 96-wells plates for three days, then established individual spheroids were combined in a single well for additional three days to allow for forming a heterotypic lung metastasis mimic. Composite mimics were then exposed to either DMSO or 1 nM panobinostat for two days. Fluorescent images from unfixed living spheroids were captured directly in the round-bottom 96-well plates with EVOS M7000 (Thermo Fisher). Images were further processed by extracting fluorescence signals of lung metastasis mimics from background and pseudo-colorize them using ImageJ (https://imagej.nih.gov/ij/).

### Combination assays

The synergistic potential of two-drug combinations was investigated by MTT-based cell viability measurements of tumor cells treated with pairwise combinations of cisplatin, doxorubicin and panobinostat in a 4 × 4-checkerboard concentration matrix format for 48h. Synergy landscapes and scores were obtained via SynergyFinder + web-tool [15] by applying the highest single-agent statistical reference model.

### In vitro and in vivo simulation of a combination trial

The three-week protocol of the SIOPEL 4 induction treatment for patients with metastatic HB [3] was scaled down into a one-week scheme for the cell culture setting. Briefly, cells were exposed to 250 nM of cisplatin at day 1, a combination of 250 nM cisplatin and 100 nM doxorubicin at day 3, 100 nM doxorubicin 6h later, and again 250 nM cisplatin at day 5, with media changes at day 3 and 5. For the combination therapy, 1 nM panobinostat was added on this SIOPEL 4 schedule on day 1, 3 and 5. Monotherapies and other two-drug combinations followed the same timing and dosing by omitting the respective drugs. Therapeutic response was measured by MTT-based cell viability assays.

In vivo testing was carried out by biogem (Ariano Irpino, Italy) according to the Italian Decree (08/2023-UT). In brief, 6–8 weeks old female CD1 nude mice (RRID:IMSR_CRL:086, Charles River Laboratories, Wilmington, MA, USA) were inoculated subcutaneously with 10 × 10^6^ PDX303 cells in 100 µl PBS and divided into 3 experimental groups of 7 animals using simple randomization when the mean tumor volume reached approximately 50 mm^3^. Because this was a pilot study, a formal power calculation was not required. Group 1 received drug vehicle, group 2 cisplatin (2 mg/kg) and doxorubicin (2 mg/kg), and group 3 the combination of cisplatin (2 mg/kg), doxorubicin (2 mg/kg) and panobinostat (7.5 mg/kg). Cisplatin and doxorubicin were administered by intravenous injection using PBS as vehicle, panobinostat dissolved in 6.25% DMSO/5% dextrose/0.03 M lactic acid was injected intraperitoneally. Tumor growth was measured by caliper two times a week, body weight determined once a week, and physical appearance, behavior, and clinical changes of mice checked every day by an experimenter blinded to injection condition and experimental cohort. Tumor volume was calculated according to the formula: (length (mm) × width (mm)^2^)/2.

### Statistical analysis

Statistical analyses were performed with GraphPad Prism 8 (RRID:SCR_002798) by displaying data as mean ± standard error of the mean (SEM) or standard deviation (SD). For all assays, two group comparisons were analyzed using unpaired Student’s t test. For survival analyses, Kaplan–Meier method with Mantel-Cox testing was performed. Correlation analyses were done with two-tailed Pearson r using a confidence interval of 0.95. Tumor growth rates of treated mice were performed using two-way ANOVA. *P*-values < 0.05 were considered significant for all analyses.

## Results

### Drug prioritization identifies panobinostat as a new drug for high-risk hepatoblastoma

**Fig. 1:**
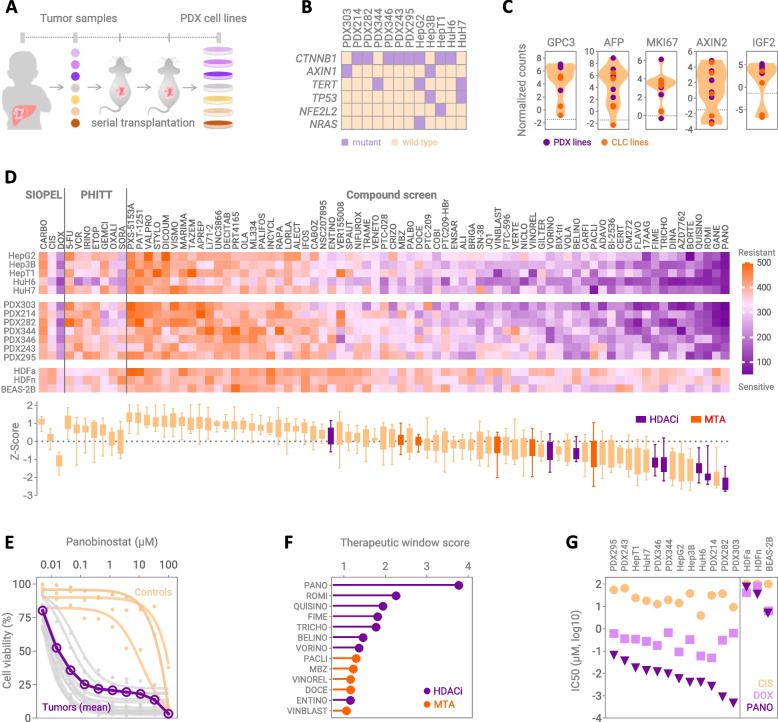
Drug prioritization strategy using a pediatric liver cancer testing platform. **A** Schematic workflow representing the establishment of hepatoblastoma patient-derived xenograft (PDX) models. **B** Mutational status and **C** RNA expression levels of marker genes in the tumor cells lines of the platform. Dashed lines represent the mean expression level of normal liver tissue. **D** Heatmap displaying the response towards drug candidates tested on the platform, as evidenced by area under the curve (AUC) values from sensitive (purple) to resistant (orange). Corresponding Z-scores of each drug candidate are shown depicting the overall efficacy of the tested drugs on all cell lines, with boxes and whiskers representing the 25th to 75th percentiles and smallest to largest values, respectively. HDAC inhibitors (HDACi) and microtubule-targeting agents (MTA) are highlighted in purple and orange, respectively. **E** Response curves displaying the cell viability upon 10 increasing concentrations of panobinostat, ranging from 5 nM to 100 µM. Each curve represents an individual cell line, with tumor models in grey and healthy controls in orange. Mean response of all tumor models is shown in purple, and each dot represents the mean value of two independent experiments with duplicate measurements. **F** Lollipop plot showing the therapeutic window score of HDACi (purple) and MTA (orange) compounds. **G** Half-maximal inhibitory concentrations (IC50) of cisplatin (CIS), doxorubicin (DOX) and panobinostat (PANO) in tumor models and non-cancerous controls. PHITT, Pediatric Hepatic International Tumor Trial; SIOPEL, Childhood Liver Tumors Strategy Group; CTNNB1, beta catenin; AXIN1, Axin 1; TERT, telomerase reverse transcriptase; TP53, tumor protein p53; NFE2L2, nuclear factor erythroid 2-related factor 2; NRAS, neuroblastoma Ras viral oncogene homolog; GPC3, glypican 3; AFP, alpha fetoprotein; MKI67, marker of proliferation Ki-67; AXIN2, Axin 2; IGF2, insulin like growth factor 2

In order to identify a therapeutic option for patients with metastatic HB, we have set up a drug testing platform comprised of seven cell culture models generated from serially transplanted PDX tumors (Fig. 1a), five established liver cancer cell lines alongside neonatal and adult skin fibroblasts as well as normal lung epithelial cells as healthy controls. Our tumor model collection covered high-risk features of HB such as lung metastases, intrahepatic relapse, age ≥ 3 years, PRETEXT IV, embryonal histology, vascular invasion, multifocal growth, transitional liver cell tumor (TLCT) differentiation, recurrent disease and poor outcome (Suppl. Table 1). Molecularly, all tumor models exhibit common driver mutations in *CTNNB1* and *AXIN1*, with TLCT models showing the characteristic *TERT* mutation, and some models progression-associated alterations in the *NFE2L2* and *NRAS* genes (Fig. 1b). RNA sequencing demarcated tumor models from normal liver cells by displaying high levels of the HB markers GPC3, AFP and IGF2, the proliferation marker Ki67, and the Wnt signaling target AXIN2 (Fig. 1c). Of note, individual models are reminiscent of Wnt non-activated, AFP negative, low proliferating, and/or IGF2 non-activated tumors, subtypes known from comprehensive genomic studies to occasionally occur in HB [7, 16, 17].

This representative drug testing platform was used to determine the therapeutic efficacy of 78 compounds (Suppl. Table 2). Except for doxorubicin, all currently applied standard-of care drugs showed only moderate efficacy in inhibiting the cell viability of high-risk HB models, as evidenced by area under the curve (AUC) values calculated from drug-response to ten increasing drug concentrations (Fig. 1d). Although most new compounds on the testing platform had only a minimal or moderate effect, we identified the group of microtubule-targeting agents (paclitaxel, vinorelbine, vinblastine, docetaxel, and mebendazole) as well as histone deacetylase (HDAC) inhibitors (panobinostat, rhomidepsin, quisinostat, fimepinostat, belinostat, and vorinostat) to be enriched in the most effective drugs displaying very low Z-scores (Fig. 1d). However, the very best candidate drug tested was the multi HDAC inhibitor panobinostat, which showed an extremely high tumor-suppressive effect throughout all HB models, while leaving healthy control cells unaffected (Fig. 1d). Moreover, the comparison of the drug inhibition curves between tumor models and healthy cells revealed a wide therapeutic window of panobinostat, which will allow for a dose optimization design in the clinical setting (Fig. 1e), while other drugs of these two groups showed much lower therapeutic window scores (Fig. 1f). By calculating the half-maximal inhibitory concentrations (IC50) between the individual HB models we revealed that panobinostat reduced viability at nanomolar levels > 1,000-times and > 10-times lower than cisplatin and doxorubicin, respectively (Fig. 1g; Suppl. Figure 1). Most convincingly, the anti-tumoral effect of panobinostat has already been validated on HepG2 tumors in mice [18] and its safety established in children [19]. Altogether, we have identified panobinostat to be the most promising drug to bring forward to the new clinical trial.

### Transcriptional upregulation of HDAC genes is associated with metastatic disease

**Fig. 2:**
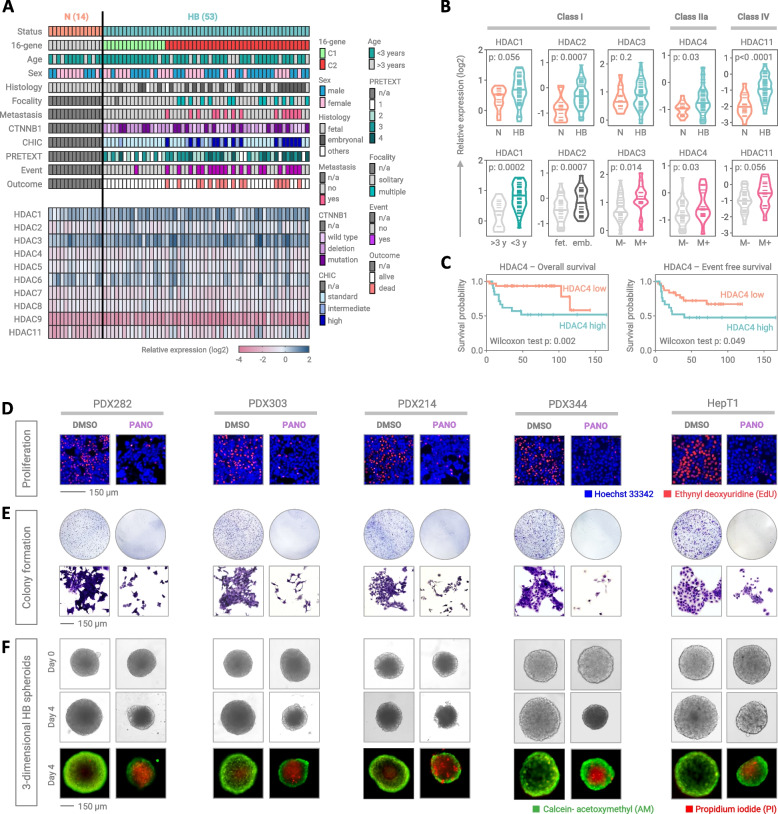
Histone deacetylase expression in hepatoblastoma patients and panobinostat effects in vitro. **A** Color-coded clinical (upper panel) and transcriptional (lower panel) characterization of patients with hepatoblastoma (HB) with corresponding normal liver tissue (N) enrolled in the JPLT-2 trial (data were retrieved from the GSE131329 data set). Relative RNA expression levels of histone deacetylases (HDACs) were calculated by normalizing the fragments per kilobase of transcript per million mapped reads of candidate genes with the housekeeping gene TATA-box binding protein. **B** Comparison of HDAC expression levels between normal liver (N) and hepatoblastoma (HB) samples as well as between different clinical characteristics. Individual values and the mean of the group are given as short and long lines, respectively. The student’s t test was applied to calculate significances between the groups. **C** Kaplan–Meier curves displaying overall and event free survival probabilities of 53 HB patients of the JPLT-2 trial with low (*n* = 21) and high (*n* = 32) HDAC4 expression. Wilcoxon test was used to calculate significance. **D** Short-term growth of proliferating tumor cells upon vehicle (DMSO) or 1 nM panobinostat (PANO) for 24h, as detected by ethynyl deoxyuridine staining (red) on Hoechst 33,342-counterstained nuclei (blue). **E** Long-term growth of hepatoblastoma cells exposed to DMSO or 1 nM PANO for 7 days, as monitored by colony formation assay. Representative crystal violet-stained wells (upper panel) and magnified views of single colonies (lower panel) are demonstrated. **F** Three-dimensional growth of established HB tumor spheroids after exposure to DMSO or 1 nM PANO for 4 days, given as microscopic brightfield images (two upper panels). The bottom panel depicts spheroids after live/dead staining with calcein-AM (green) and propidium iodide (red), respectively. 16-gene, subtype according to the 16-gene signature; CTNNB1, beta catenin; CHIC, risk group according to the Children's Hepatic Tumors International Collaboration; PRETEXT, pre-treatment extent of tumor; n/a, not applicable

Panobinostat is a pan-inhibitor of HDACs and response to HDAC inhibition has been reported to be predictable by the expression levels of HDACs themselves [20]. By analyzing RNA abundance of HDACs in the clinically well annotated cohort of HB patients enrolled in the JPLT-2 trial [9], we detected a substantial expression of all HDACs in normal liver as well HB samples, except for HDAC9 (Fig. 2a). Interestingly, HDAC1, HDAC2, HDAC4, and HDAC11 showed a significant upregulation in tumor compared to normal liver tissue (Fig. 2b). We next wanted to see if expression of these HDACs was associated with specific clinicopathological characteristics. Significantly higher RNA expression levels were found in tumors of patients younger than 3 years (HDAC1), with embryonal histology (HDAC2), and most prominently metastases (HDAC3, HDAC4, and HDAC11) (Fig. 2b). Of clinical importance, high HDAC4 expression was associated with poor event-free and overall survival (Fig. 2c). Collectively, these data suggest that patients with metastatic HB and/or poor outcome could benefit most from a treatment with panobinostat.

### Panobinostat massively reduces the growth properties of hepatoblastoma

**Fig. 3:**
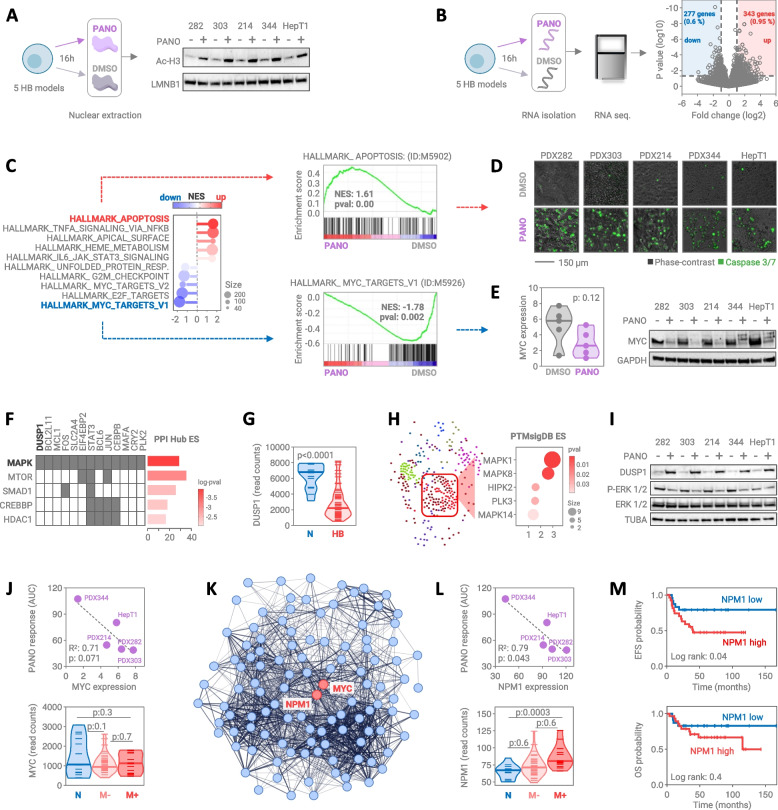
Molecular consequences of panobinostat treatment. **A** Western blot analysis of five hepatoblastoma models showing acetylated histone 3 (AcH3) protein levels after exposure to vehicle (DMSO) or 1 nM panobinostat (PANO) for 16h. Lamin B1 (LMNB1) served as nuclear loading control. **B** Volcano plot of expressed genes after RNA sequencing demonstrating significantly up- (red) and down-regulated (blue) genes in five hepatoblastoma models following 16h incubation with DMSO or 1 nM PANO. Dashed lines represent log2 fold increase and *p* < 0.05 significance thresholds. **C** Gene set enrichment analysis of RNA sequencing data showing positively (red) and negatively (blue) enriched HALLMARK gene sets (left panel), given as normalized enrichment scores (NES) and number of genes involved in the respective gene sets (size). The two top ranked HALLMARK gene sets are shown as individual enrichment plots (right panel). **D** Apoptotic cells detected by fluorescent staining of active caspase 3 and 7 substrates (green). Fluorescent images of tumor lines were captured after 24h incubation with DMSO or 1 nM PANO. **E** MYC expression in tumor cells treated with DMSO or 1 nM PANO on the RNA (left panel) and protein level (right), as evidenced by RNA sequencing and Western blot, respectively. **F** Enrichr-generated clustergram of significantly upregulated genes upon PANO exposure given as protein–protein-interaction Hub enrichment scores (PPI Hub ES) for enriched molecular categories and color-coded logarithmic p values. **G** RNA levels of DUSP1 in 53 patients with hepatoblastoma (HB) and corresponding normal liver tissue (N) of the JPLT-2 trial. The student’s t test was applied to calculate significance. **H** Subnetwork module map of significantly upregulated genes upon PANO exposure showing enriched kinases after kinase enrichment analysis utilizing the post-translational molecular signature database (PTMsigDB) and weighted gene co-expression network analysis (WGCNA) (left panel). The bubble plot shows top scoring kinases (right panel), with the size of bubbles representing the number of involved genes and the color scale indicating significance. **I** Western blot analysis demonstrating the expression levels of DUSP1, ERK 1/2, and phospho-ERK 1/2 (Thr202/Tyr204) proteins in hepatoblastoma models treated with DMSO or 1 nM PANO for 16h. Alpha tubulin (TUBA) was used as loading control. **J + L** Correlation between response of hepatoblastoma models towards PANO, given as area under the curve (AUC) and RNA sequencing-derived MYC and NPM1 expression, with R2 correlation coefficients and p values calculated by two-tailed Pearson test and linear regressions given as dashed lines (upper panels). Violin plot demonstrating the RNA levels of MYC and NPM1 in patients with hepatoblastoma (HB) and corresponding normal liver tissue (N) of the JPLT-2 trial, stratified into groups of 14 normal liver samples (N), 39 non-metastatic (M-) and 14 metastatic patients (M +). Significance was calculated by Student’s t test (lower panel). **K** STRING protein–protein interaction network of the significantly downregulated genes of the HALLMARK_MYC_TARGETS_V1 gene set (111 out of 200 genes) upon PANO treatment, highlighting connections and neighborhood of the involved proteins. The nodes represent proteins, the edges indicate both functional and physical protein associations, and the line thickness indicates the strength of data support. **M** Kaplan–Meier curves displaying event-free survival (EFS) and overall survival (OS) probabilities for patients with hepatoblastoma of the JPLT-2 trial with either high (*n* = 28) or low (*n* = 26) *NPM1* expression. The log-rank Mantel-Cox test was used to calculate significance

In order to investigate the cellular consequences of panobinostat treatment, we applied the drug as monotherapy given at IC50 levels to five selected high-risk HB models and evaluated various growth-associated properties. The proliferative capacity of tumor cells, retrieved from the incorporation of a labeled nucleoside analog during the S phase of actively dividing cells, was dramatically impaired upon panobinostat treatment for 24 h (Fig. 2d). Congruently, long-term survival of tumor cells over a time of 7 days was almost completely blocked when HB models were propagated in colony formation assays (Fig. 2e). Most importantly, when HB models were grown as three-dimensional tumor spheroids, panobinostat treatment resulted in irregular shaped and size-diminished structures, indicative of compromised viability and growth (Fig. 2f). Accordingly, life/death staining of respective spheroids revealed strong accumulation of dying cells in the center of panobinostat-treated models (Fig. 3c). These data clearly underscore the capability of panobinostat in the almost complete growth abrogation of HB cells.

### Myc signaling as the priority target of panobinostat in hepatoblastoma

HDAC inhibition is anticipated to prevent deacetylation of histones, thereby leading to widespread changes of transcriptional activities in cancer cells [21]. By applying Western blot and RNA sequencing analyses, we could indeed prove that panobinostat treatment resulted in increased levels of acetylated histone 3 (Fig. 3a) and differential expression of 620 genes (Fig. 3b), when integrating the response in five different HB models. Gene set enrichment analysis of the RNA sequencing data revealed apoptosis as the most prominent molecular consequence triggered by panobinostat in pediatric liver cancer cells (Fig. 3c). Accordingly, we could validate this hallmark of cancer by staining for cleaved caspase 3 and 7 in panobinostat treated cells (Fig. 3d). More importantly, panobinostat most prominently mediated transcriptional downregulation of MYC target genes, although MYC itself was mainly unchanged on the RNA level (Fig. 3e). Instead, panobinostat treatment led to a dramatic decrease in MYC protein levels, thereby implying that posttranslational changes of MYC might be responsible for the impeded downstream signaling of MYC detected upon RNA sequencing (Fig. 3e).

In order to identify candidates that modulate MYC activity, we performed protein–protein interaction analysis of transcriptionally upregulated genes and identified mitogen-activated protein kinase (MAPK) signaling as the most prominently enriched category (Fig. 3f). One important protein of this category is the dual specificity phosphatase 1 (DUSP1), which is known to regulate activity of MAPKs by dephosphorylation of their tyrosine and threonine residues [22]. Interestingly, DUSP1 is heavily downregulated in tumors of HB patients (Fig. 3g). Kinase enrichment analysis of upregulated genes showed that MAPK1 might be the main target of DUSP1 (Fig. 3h). Indeed, Western blot experiments clearly indicated that panobinostat leads to increased protein levels of DUSP1, subsequently resulting in the dephosphorylation of the ERK2 protein, which is encoded by MAPK1 (Fig. 3i). ERK/MAPK has been described to act as the pioneering kinase to phosphorylate and thereby stabilize MYC protein [23]. Altogether, these results suggest that panobinostat decreases MYC activity through DUSP1-mediated downregulation of ERK/MAPK.

We next wondered whether MYC expression could indicate responsiveness of HB cells to panobinostat. Indeed, high MYC expression levels in the HB models were highly correlated with AUC values of treatment (Fig. 3j). However, MYC expression retrieved from transcriptomic data of HB patients indicated that MYC cannot be used as a predictive marker, as it is nearly unchanged between tumor and normal liver tissue. We therefore generated a protein–protein interaction network to identify a putative surrogate marker that could substitute MYC in being indicative for MYC activation in hepatoblastoma. Nucleophosmin 1 (NPM1), a transcriptional target of MYC [24], showed the strongest and closest association with MYC (Fig. 3k). Interestingly, NPM1 expression was significantly higher in tumor than in normal liver tissue, especially in patients with metastatic disease, and correlated well with response to panobinostat (Fig. 3l). By analyzing survival data of HB patients, we could demonstrate that high NPM1 expression was associated with poor event-free survival, and showed a trend towards poor overall survival (Fig. 3m). Thus, our data define NPM1 as a putative marker to predict response to panobinostat and outcome of HB patients, especially those with metastatic disease.

### Panobinostat is highly efficacious in MYC-driven liver tumor cells

**Fig. 4:**
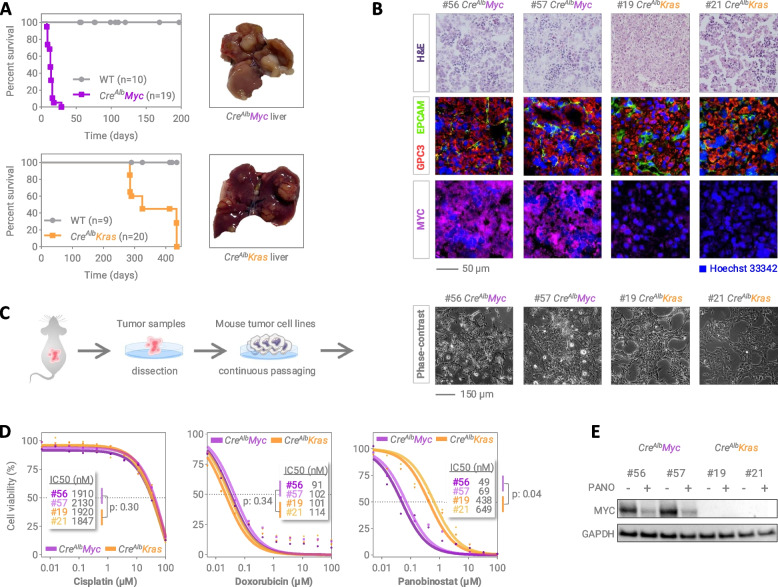
Susceptibility of MYC-activated tumor cells to panobinostat treatment. **A** Kaplan–Meier survival curves of Cre^Alb^Myc and Cre^Alb^Kras mice (left), and representative liver images demonstrating the lesions (right). **B **Representative cryosections from Cre^Alb^Myc and Cre^Alb^Kras cohorts stained with hematoxylin and eosin (H&E) (top), fluorescently stained for the hepatoblastoma markers glypican 3 (GPC3; red) and epithelial cell adhesion molecule (EPCAM; green) (middle), and the MYC proto-oncogene (MYC, magenta) (bottom). **C **Schematic overview of generating mouse tumor cell lines from dissected tumors (left) and microscopic images of the established models (right). **D **Cell viability curves and IC50 values demonstrating the response of the Cre^Alb^Myc (purple) and Cre^Alb^Kras (orange) mouse tumor cell lines towards increasing concentrations of cisplatin, doxorubicin, and panobinostat. **E **Western blot analysis highlighting the expression level of MYC protein in Cre^Alb^Myc and Cre^Alb^Kras mouse models treated with DMSO or 1 nM PANO for 16h. Glycerinaldehyd-3-phosphat-dehydrogenase (GAPDH) was used as loading control

In order to investigate if the anti-tumoral effect of panobinostat is selective on MYC overexpressing hepatoblastoma or liver cancer in general, we phenocopied both scenarios in respective liver cancer mouse models. Crossing *LSL-MYC* with Cre^Alb^ mice drives constitutive overexpression of the MYC oncogene in the liver and leads to the development of liver tumors in all mice within 4 weeks (Fig. 4a). These tumors resemble hepatoblastoma based on their early onset, their characteristic histology and the expression of specific markers such as GPC3 and EPCAM, but most importantly high MYC expression (Fig. 4b). In contrast, crossing *LSL-Kras*^*G12D*^ with Cre^Alb^ mice leads to permanent activation of oncogenic Kras in the mouse liver and results in liver tumor development in all mice within 14 months (Fig. 4a). These tumors were diagnosed as hepatocellular carcinoma with the expression of GPC3 and EPCAM, but lacking MYC overexpression (Fig. 4b). From each tumor model we established two permanent cell lines (Fig. 4c) and performed viability assays upon panobinostat treatment. Strikingly, the response of MYC-expressing hepatoblastoma cells to panobinostat was 10-times stronger than the one of Kras-driven tumor cells, whereas the antitumor effect of cisplatin and doxorubicin were comparable in both models (Fig. 4d). Most importantly, MYC protein levels in the MYC model were dramatically decreased by panobinostat (Fig. 4e). Altogether, these results clearly indicate that panobinostat selectively impacts MYC-expressing hepatoblastoma.

### Therapy response in mimics of lung metastasis

**Fig. 5:**
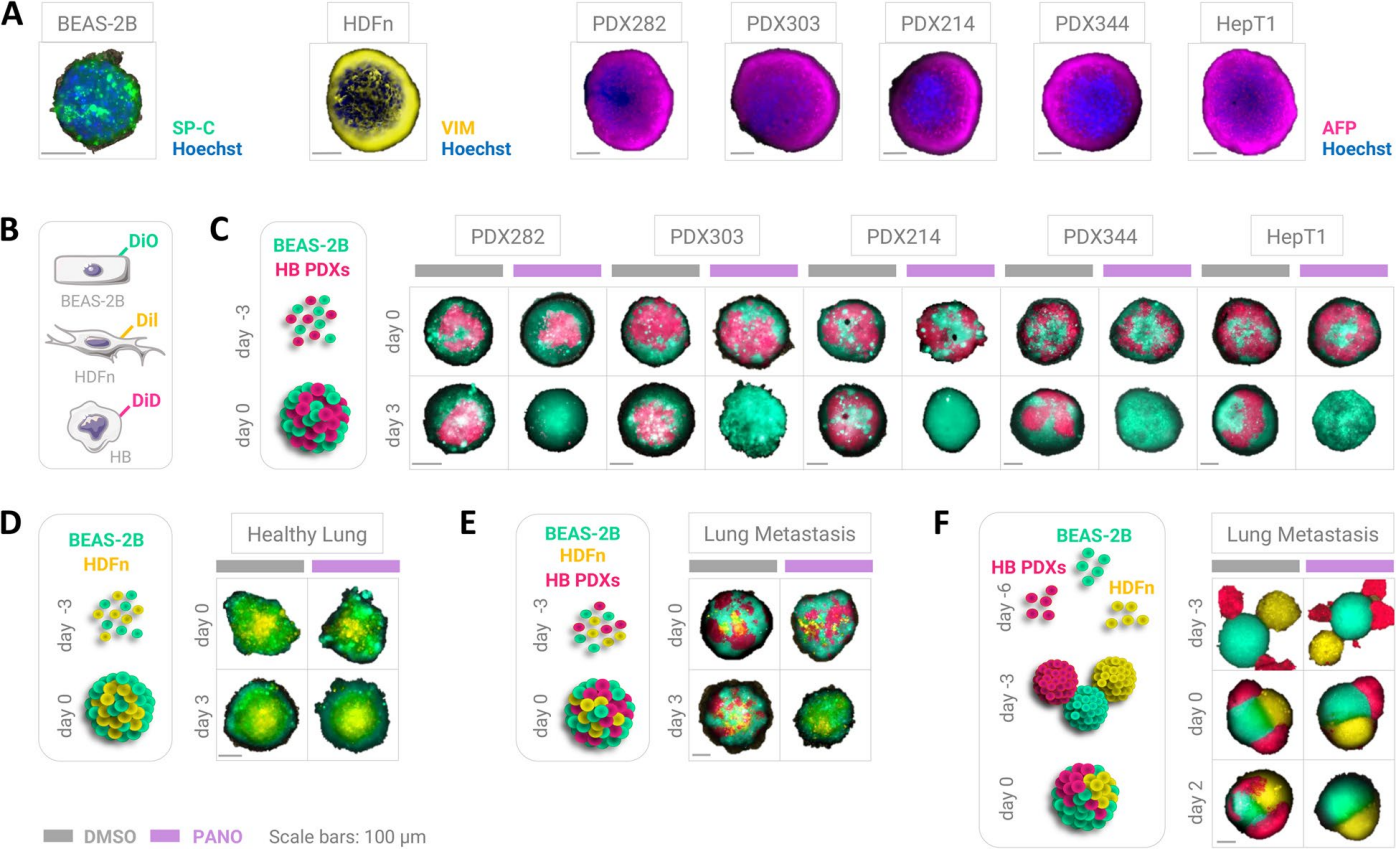
Panobinostat treatment of lung metastasis mimics. **A** Immunofluorescence images of individual spheroids of lung epithelial cells (BEAS-2B), neonatal skin fibroblasts (HDFn) and hepatoblastoma models stained for the airway epithelial cell marker surfactant C (SP-C), the mesenchymal marker vimentin (VIM) and the hepatoblastoma marker alpha fetoprotein (AFP), respectively. **B **BEAS-2B, HDFn and HB cells were labeled with the tracking dyes Vybrant DiO, Dil and DiD, respectively. **C **Basic lung metastasis spheroid model consisting of lung epithelial cells (green) and tumor cells (pink) treated with vehicle (DMSO) or 1 nM panobinostat (PANO). **D **Normal lung spheroid model with lung epithelial cells (green) and fibroblasts (yellow). **E **Heterotypic lung metastasis spheroid model comprised of lung epithelial cells (green), fibroblasts (yellow), and tumor cells (pink) after DMSO or 1 nM PANO treatment. **F **Infiltrative lung metastasis spheroid model generated from individual spheroids of lung epithelial cells (green), fibroblasts (yellow), and tumor cells (pink) upon exposure to DMSO or 1 nM PANO. Time of spheroid formation and treatment as well as scale bars are indicated

In order to predict the therapeutic potential of panobinostat on lung metastases, we have produced different three-dimensional heterotypic metastasis models comprised of dermal fibroblasts, epithelial lung cells, and five different PDX tumor cells, all of which expressed cell-specific marker proteins when grown as monotypic spheroids (Fig. 5a). Applying panobinostat to spheroids formed through co-culturing of differentially labeled (Fig. 5b) tumor cells together with lung cells resulted in the complete depletion of tumor cells from these three-dimensional models (Fig. 5c), whereas spheroids of healthy fibroblasts and lung cells were unaffected (Fig. 5d). The selective eradication of tumor cells could be similarly reached in heterotypic models comprised of all three cell types (Fig. 5e; Suppl. Figure 2). Even when the individual cell types preformed homotypic spheroids that were subsequently allowed to fuse into each other in order to simulate tumor infiltrative growth, panobinostat was able to eliminate the tumor compartment within two days (Fig. 5f; Suppl. Figure 2). These simulation experiments convincingly show the potential of using panobinostat for treating patients with metastatic hepatoblastoma.

### Simulating a clinical trial with panobinostat on the SIOPEL 4 backbone

**Fig. 6:**
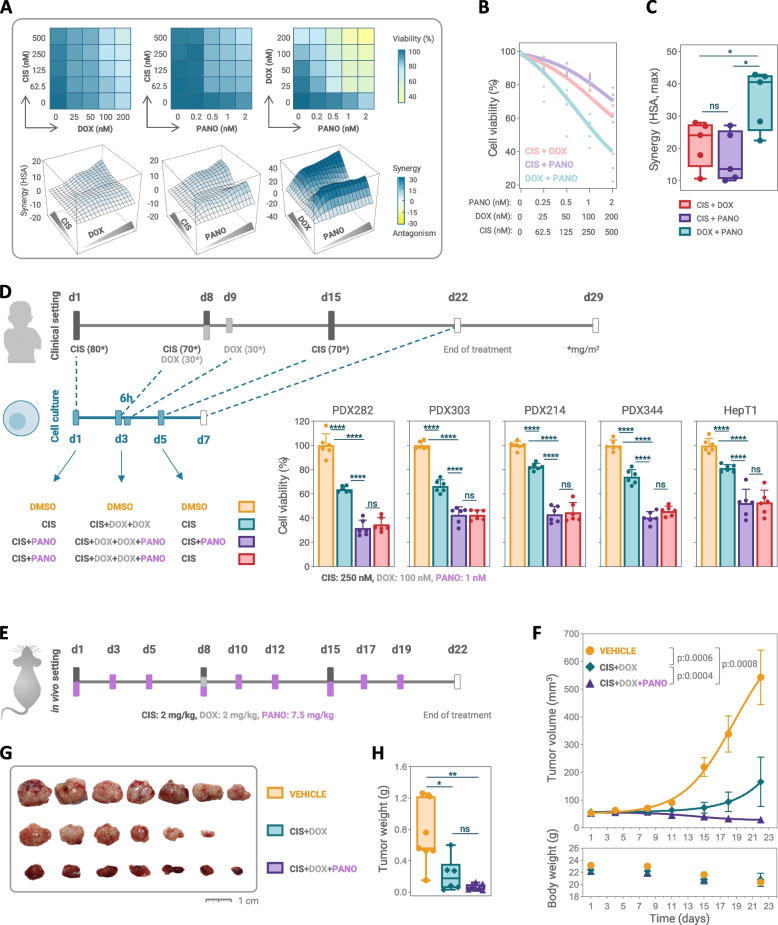
Simulation of a clinical trial in vitro and in vivo. **A **Representative cell viability-matrix (top) and 3-dimensional synergy landscapes (bottom) for pairwise-drug combination of cisplatin (CIS), doxorubicin (DOX) and panobinostat (PANO) with four increasing concentrations demonstrated in PDX214 cells. **B **Summarized cell viability curves of five hepatoblastoma models with sensitivity towards two-drug combination of CIS, DOX and PANO for indicated concentrations. Lines represent the mean of two independent experiments with duplicate measurements, each dot represents an individual tumor model. **C **Summarized maximum synergy scores of five hepatoblastoma models corresponding to given cell viability curves upon two-drug combination of CIS, DOX and PANO. The dots represent individual tumor cell lines, whiskers show min-to-max values and horizontal lines indicate the median. **D **Schematic overview of one cycle of the SIOPEL 4 induction regimen and its adaptation to the in vitro setting. Bar graphs show the cell viability for determined drug combinations, with the mean ± SD of two independent experiments in triplicates. Statistics were calculated using a two-tailed unpaired Student’s *t* test, with ns = not significant, *p < 0.05, **p < 0.01, ***p < 0.001, ****p < 0.0001. **E** Dosing protocol of the in vivo drug testing in the PDX303 xenograft mouse model. **F **Tumor growth (upper panel) and body weight changes (lower panel) in mice treated with either CIS + DOX, CIS + DOX and PANO, or vehicle. Values correspond to mean tumor volumes and mean body weights ± SEM, and differences to vehicle were calculated using two-way Anova. **G **Pictures and **H **weights of dissected tumors at day 22

In order to investigate the likely beneficial impact of combining panobinostat with the SIOPEL 4 induction medications cisplatin and doxorubicin [3], we tested five HB models in a high accuracy 4 × 4-checkerboard with dose escalation of a pairwise-drug combination matrix. Each two-drug combination led to a reduction of cell viability in all HB models and corresponding three-dimensional synergy landscapes revealed high synergies of the combination therapies (Fig. 6a, Suppl. Figure 3). Most importantly, the panobinostat/doxorubicin combination showed the strongest effect on cell viability and two similar high synergy ridges, with one at very low nanomolar levels (Fig. 6b). Plotting response rates only for the doubling drug concentrations indicated that the effect on cell viability is dose-dependent for all drug combinations, but highest for panobinostat/doxorubicin (Fig. 6b, Suppl. Figure 4). Accordingly, calculated synergy scores were significantly higher for the panobinostat/doxorubicin combination than for the other two combinations (Fig. 6c, Suppl. Figure 4).

We next wanted to simulate a clinical trial with panobinostat built onto the SIOPEL 4 backbone in the cell culture setting (Fig. 6d). By scaling the 3-week clinical protocol down to a 1-week in vitro treatment in cell culture, we could see a significant reduction of cell viability by using the SIOPEL 4 medications. Strikingly, the addition of panobinostat to the cisplatin/doxorubicin combination almost doubled the inhibitory effect on tumor cells (Fig. 6d). Monotherapies and two-drug combinations of cisplatin, doxorubicin and panobinostat following the same dosing schedule furthermore showed that panobinostat in combination with doxorubicin has the highest anti-tumor effect (Suppl. Figure 5). As the highest synergy can be achieved by using panobinostat in combination with doxorubicin (Fig. 6c), and knowing that the third week of the SIOPEL 4 protocol only foresees a single infusion of cisplatin, we wanted to define the anti-tumor effect of the combination treatment by omitting panobinostat at this timepoint. Of utmost significance, the strong inhibitory effect was not attenuated in the reduced combination protocol (Fig. 6d), thereby allowing for a balanced dosing of panobinostat in the clinical setting.

Ultimately, we modeled the SIOPEL 4 treatment with or without panobinostat using the subcutaneous xenograft mouse model PDX303 (Fig. 6e). Mice treated with a combination of cisplatin and doxorubicin showed significantly slower tumor growth compared to the control group (Fig. 6f). When panobinostat was added to the cisplatin/doxorubicin combination three times a week, tumor growth was further reduced, and in the second week, even regressed following concurrent doxorubicin treatment. Notably, a dose of 7.5 mg/kg panobinostat in addition to cisplatin/doxorubicin was not interfering with continuation of the therapy, as no significant weight loss (Fig. 6f) or other adverse effects were observed. However, the tumors dissected from the cisplatin/doxorubicin/panobinostat group had a hemorrhagic appearance (Fig. 6g), suggesting a complete response to the combination therapy. Collectively, our preclinical data clearly advocate for panobinostat to be implemented into the SIOPEL 4 protocol.

## Discussion

Hepatoblastoma patients with metastatic disease, especially those that do not respond well to first-line chemotherapies, still face a very poor prognosis, necessitating new treatment strategies tailored to more efficiently reducing tumor load in the lung. In this study, a multilayered strategy defined panobinostat as the most appropriate drug to be tested in a clinical trial in combination with the currently used SIOPEL 4 protocol [3] in patients with metastatic hepatoblastoma. This was achieved by collecting literature data on preclinically tested drugs, validating their efficacy on a comprehensive pediatric liver cancer testing platform, establishing the mechanistic background of drug-induced growth inhibition, proving the anti-cancer effect in lung metastasis mimics, and simulating an optimized treatment protocol in vitro and in vivo.

Panobinostat is a potent oral pan-histone deacetylase inhibitor that has already been used in several clinical trials in the adult and pediatric population with different schedules, presenting as a relatively safe, tolerable and efficacious drug [19, 25–29]. It is currently approved for the treatment of multiple myeloma and the subject of clinical investigations for the treatment of other cancers. Although it seems that HDAC inhibitors show higher efficacy in hematological malignancies than in solid tumors, our finding of MYC being a primary target of panobinostat in addition to its anticipated general ability to inhibit HDAC function provides a valuable rationale to use this drug for the treatment of hepatoblastoma. MYC has been described as a key marker of aggressiveness in hepatoblastoma, including invasive and metastatic disease, and its downregulation in hepatoblastoma cells impaired tumorigenesis in vivo [30]. Thus, MYC deactivation displays a promising therapeutic strategy in hepatoblastoma, as for many hematological and solid malignancies as well, but so far, MYC has been considered “undruggable” in the clinical setting [31]. We provide evidence that panobinostat inhibits tumor growth and induces apoptosis of hepatoblastoma cells by robustly downregulating MYC at the protein level. Of note, the anti-tumoral effect of panobinostat was 10-times more effective on MYC-driven than on Kras-driven liver tumor cells. In line with our results, MYC modulation has been identified as a molecular readout of HDAC inhibition in acute myeloid leukemia cells [32], and the inhibition of MYC transcription by panobinostat has recently been described in glioblastoma models as well [33]. Mechanistically, we could link MYC ablation to the decrease of its phosphorylating protein MAPK1 and the increase of DUSP1, which phosphorylates and thereby activates MAPK1. We furthermore described NPM1 as a surrogate marker for MYC activation in hepatoblastoma, which we found to be predominantly expressed in metastatic hepatoblastoma, but also in many other cancers [34]. Accordingly, we detected a strong correlation between NPM1 and MYC expression, which has also been reported for other cancers before [34]. Recent studies have underscored the strong interrelationship of NPM1 and panobinostat by identifying that CRISPR-edited acute myeloid leukemia cells with NPM1 knockout are transcriptionally mimicked by panobinostat [35] and that panobinostat and the NPM1 inhibitor avrainvillamide-analog-6 had similar effects on hippocampal cells [36]. Altogether, these observations provide ample biological rationale why panobinostat is working so efficiently in MYC/NPM1-associated hepatoblastoma cells.

It has been suggested that treatment with panobinostat may prime cancer cells to increase their sensitivity to subsequent chemotherapy [28]. The concept of HDAC inhibition to serve as a chemosensitizer is not new and was mainly attributed to the role of HDACs in promoting DNA repair [37]. HDAC inhibitors have been evaluated in combination with platinum-based drugs in many cancers, including hepatocellular carcinoma [38]. It has been described that pretreatment of human cancer cell lines with HDAC inhibitors before treatment with anticancer drugs considerably increased their cytotoxicity, especially with doxorubicin [39]. This is in line with our data showing a significantly higher cytotoxic effect and synergy with doxorubicin/panobinostat than for the cisplatin/panobinostat combination, both in vitro and in vivo. It is important to note that for establishing synergistic effects, HDAC inhibitor-elicited influences on histone acetylation have to precede the contact of the anticancer drugs with the DNA [39]. As our in vitro simulation detected a comparable efficacy when omitting panobinostat from the third week of therapy when cisplatin is given alone [3], adding reduced doses of panobinostat to the SIOPEL 4 backbone could be proposed for a clinical trial. This is especially important to allow for a better control of blood cells, as the most common side effects of panobinostat in clinical trials have been reported to be neutropenia and thrombocytopenia [19, 28, 29], which would increase the myelosuppression of the currently applied SIOPEL 4 regimen [3]. Panobinostat has been safely given to children with progressive diffuse midline glioma in a phase I trial at 10 mg/m2/dose and administered 3 times per week for 3 weeks on/1 week off [19], a schedule perfectly resembling one cycle of the SIOPEL 4 regimen [3]. Based on these findings, incorporating panobinostat into the SIOPEL 4 induction regimen in an adaptable trial design for patients with metastatic hepatoblastoma warrants clinical validation. Of note, a similar approach has just recently been described to induce cytolytic effects in hepatoblastoma PDX models in vivo by using panobinostat in combination with vincristine/irinotecan [40], which is used by the US American Children's Oncology Group to treat high-risk hepatoblastoma patients upfront [41]. This approach might be worth considering for salvage options if tumors do not respond well to panobinostat/cisplatin/doxorubicin induction therapy and/or show progression.

## Conclusions

Collectively, through a multistep prioritization process we were able to define panobinostat as the best suited drug to be used to target metastatic disease in patients with hepatoblastoma. The use of epigenetic manipulation should aim to sensitize tumor cells to the SIOPEL 4 induction treatment in order to achieve complete remission in the lung. This work further strengthens the appeal of using multifaceted in vitro testing platforms for the design of more sophisticated anticancer therapies.

## Supplementary Information

Supplementary Material 1.

## Data Availability

The datasets used and/or analysed during the current study are available from the corresponding author on reasonable request.
