# Supplementary material for: Drug prioritization identifies panobinostat as a tailored treatment element for patients with metastatic hepatoblastoma
Source: J Exp Clin Cancer Res. 2024 Nov 12;43:299. doi: 10.1186/s13046-024-03221-6 (PMC11556140; doi:10.1186/s13046-024-03221-6)
Supplement: Supplementary file 1 — Supplementary Material 1. [file 13046_2024_3221_MOESM1_ESM.pdf]

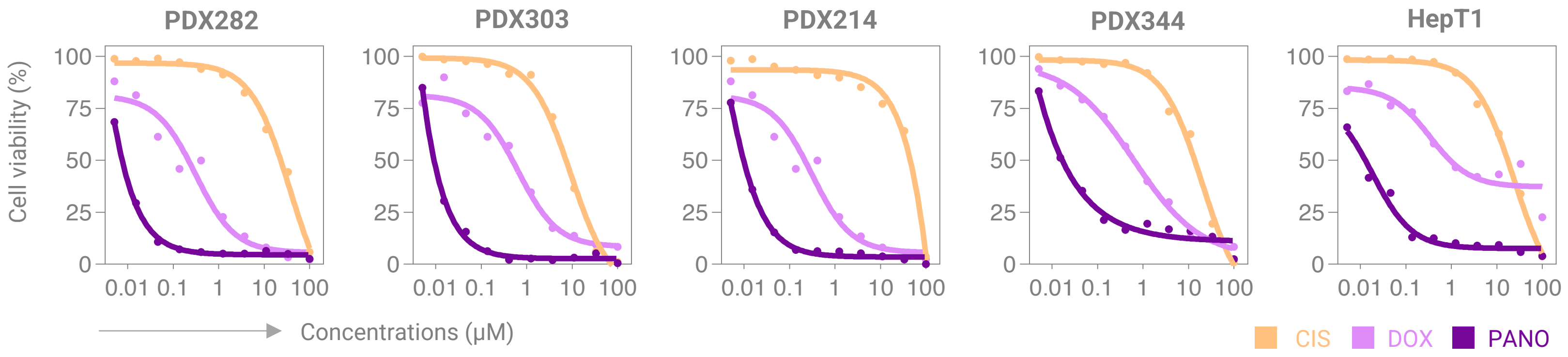

Supplementary Figure 1: Response curves of five tumor lines towards CIS, DOX and PANO, and their pairwise combinations: Cell viability curves showing cell viability upon 10 increasing concentrations of PANO, DOX and CIS.

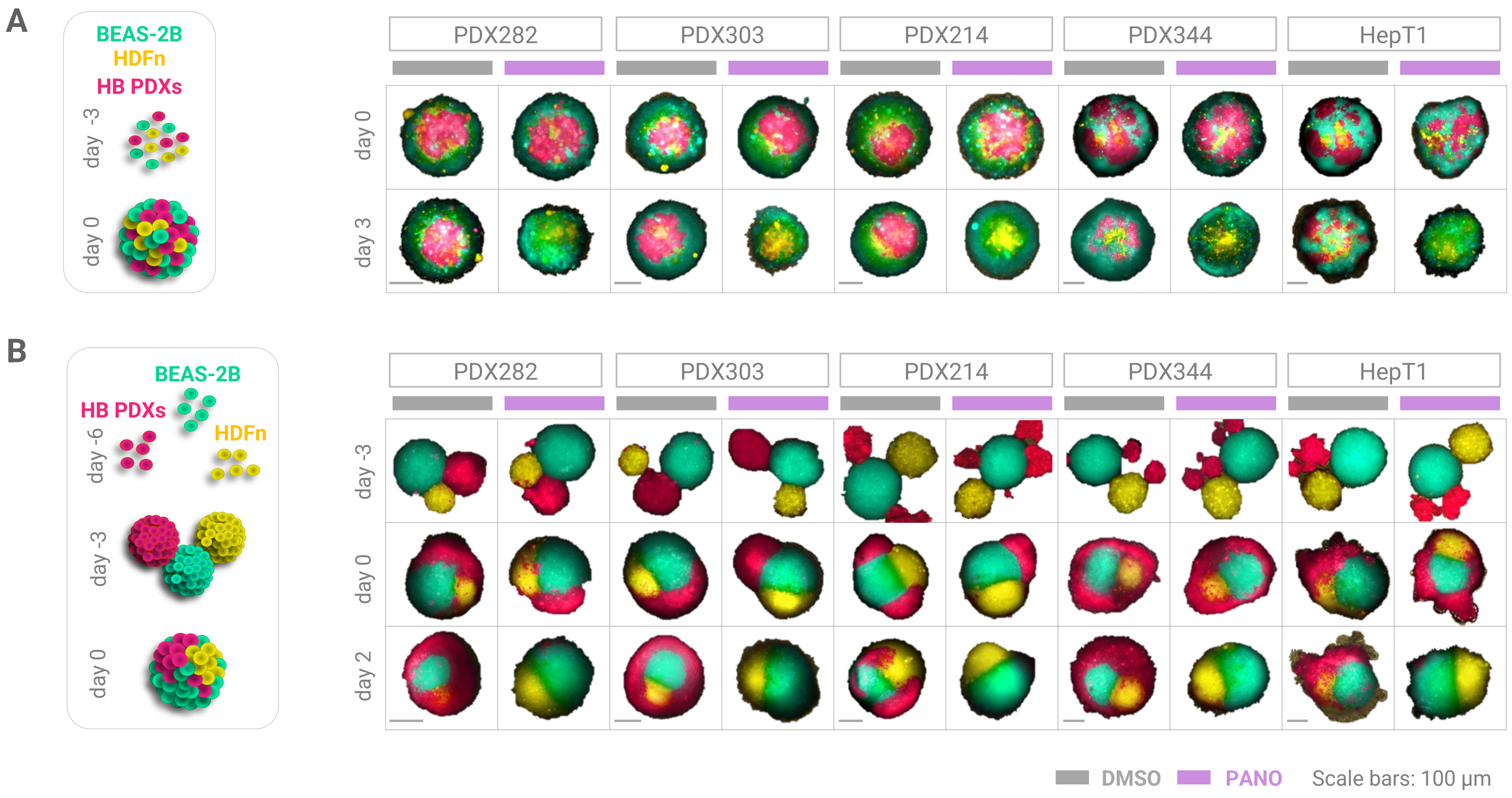

Supplementary Figure 2: Lung metastasis models with five tumor lines: Heterotypic lung metastasis mimics composed of BEAS-2B (green), HDFn (yellow) and HB tumor lines (pink). Schematic overview of the establishment of (A) mixed-spheroid-metastasis model and (B) merged-spheroid-metastasis model, and detection of tumor cells upon DMSO (grey) or PANO (purple) are displayed.

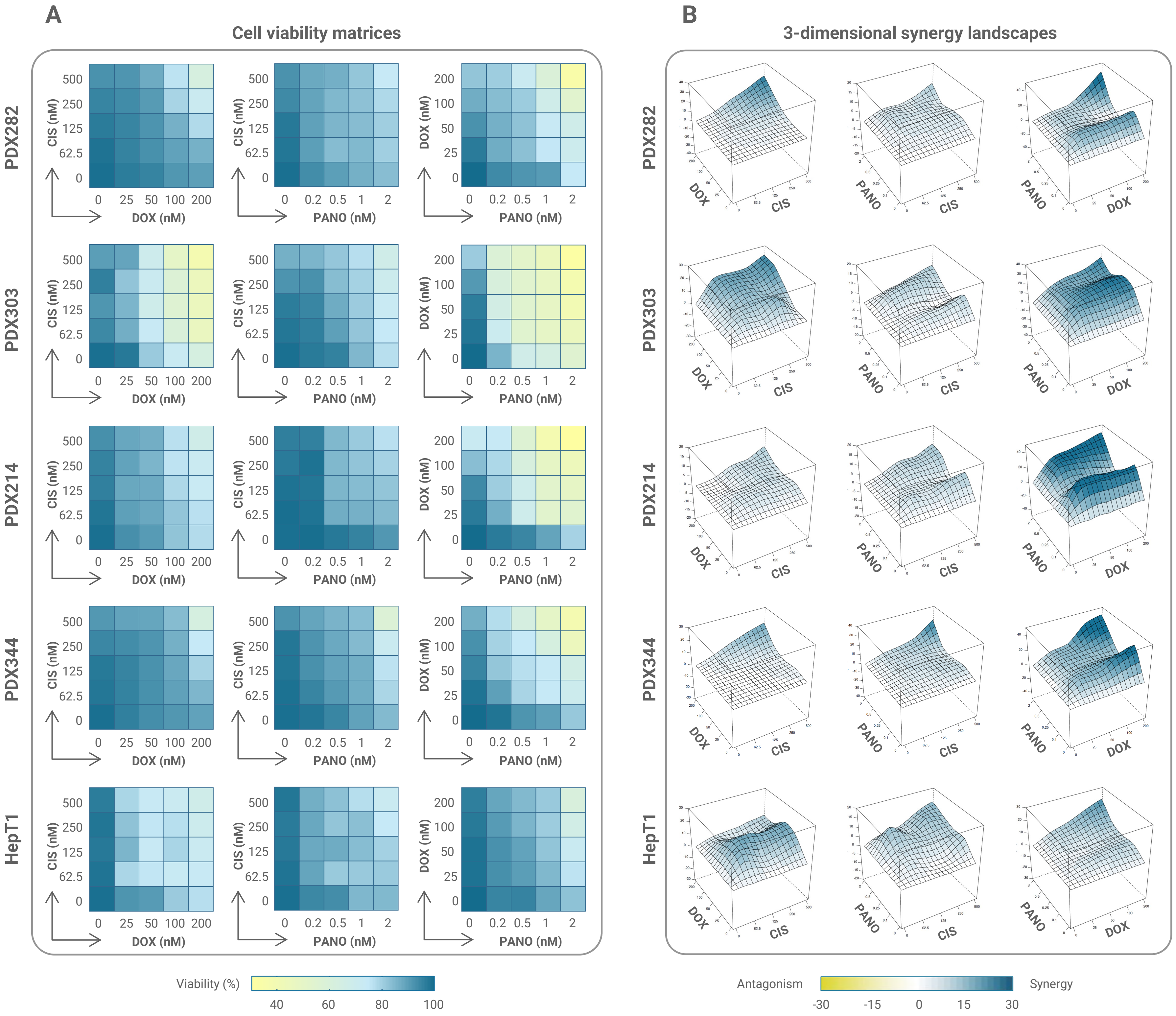

Supplementary Figure 3: Pairwise combination of CIS, DOX and PANO in five HB models: (A) Cell viability matrices for five individual HB models upon pairwise combination of CIS, DOX and PANO with four increasing concentrations for 48h. (B) Three-dimensional synergy landscapes corresponding to pairwise combinations CIS, DOX and PANO are representing the synergy distribution for all possible drug concentration combination obtained from cell viability matrices.

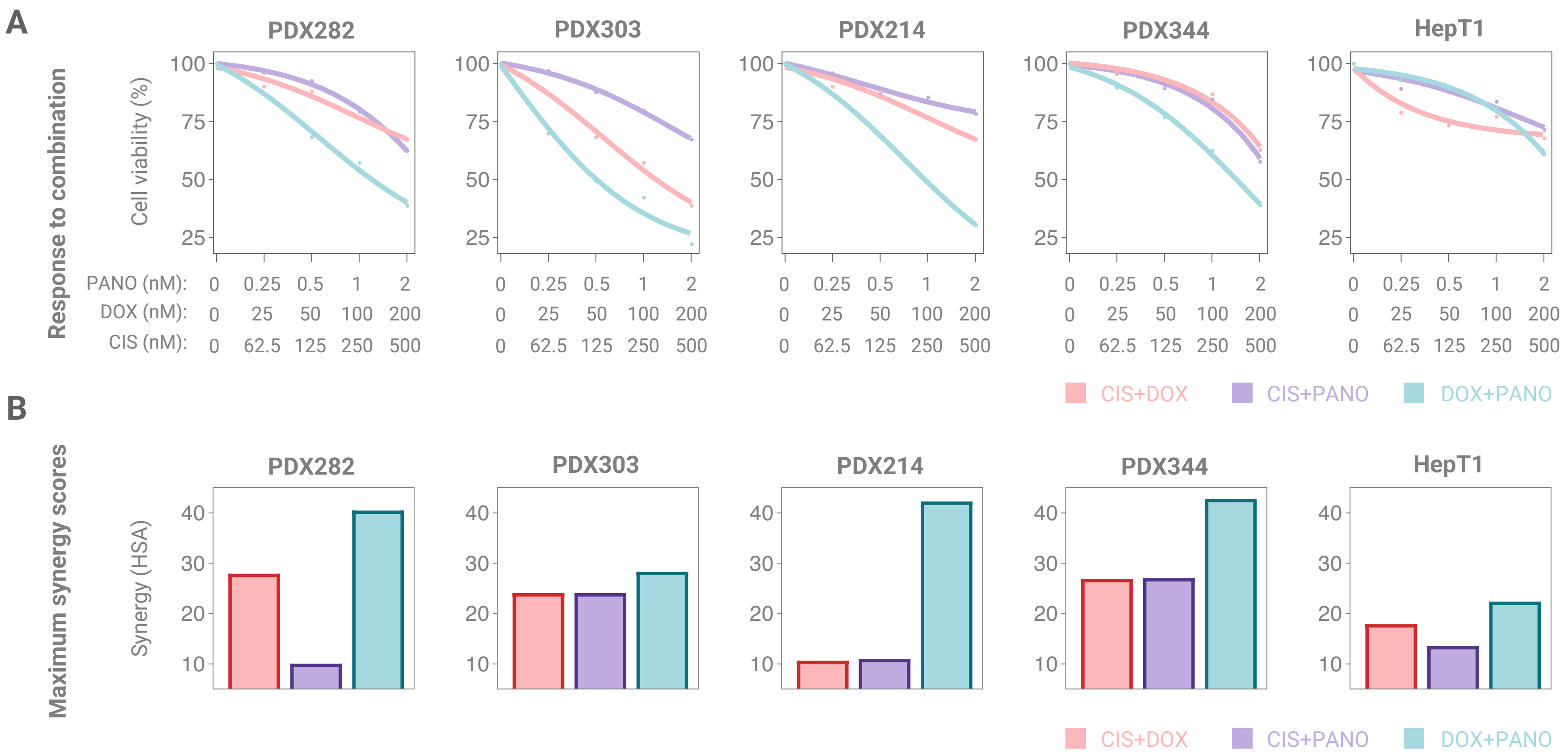

Supplementary Figure 4: Response curves and synergy scores of five tumor lines towards pairwise drug combinations (A) Sensitivity of HB tumor models upon pairwise combination of PANO, DOX and CIS with 1:2 constant escalation ratio demonstrates the cell viability in given concentrations. (B) Maximum synergy scores of HSA statistical model, by analyzing combination responses, are displayed in a bar graph.

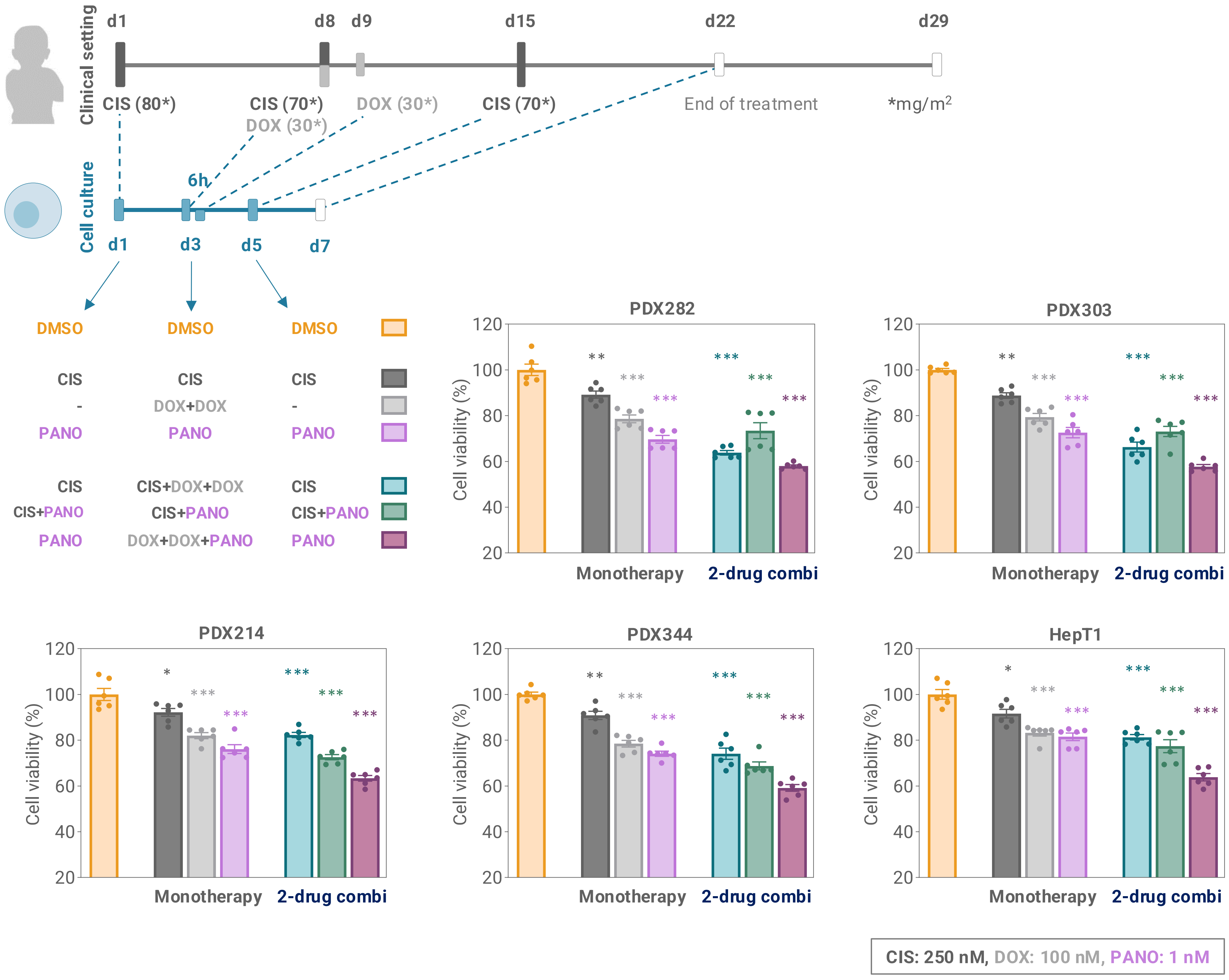

Supplementary Figure 5: Schematic representation of the combination therapy for the high-risk patients and its scaled-down translation to the cell culture setting is shown as illustrations. Bar graphs display the percentages of cell viability upon CIS, DOX and PANO monotherapy, as well as their pair-wise combination. Data is shown as means of standard error (±SEM) of two independent experiments with three replicates. Statistical significance was calculated by t-test, each group compared to DMSO control, *p<0.05 **p<0.01 ***p<0.001

Supplementary Table 1: cell lines and clinical annotations

Cell line	Supplier	Catalog no.	RRID	Culture condition	Diagnosis	Type of sample	Age (months)	Sex	PRETEXT	Main epithelial component	Vascular invasion	Multifocality	Metastasis	Outcome
HuH6	Japanese Collection of Research Bioresources, Osaka, Japan	JCRB0401	CVCL_4381	RPMI 1640 supplemented with 10% fetal bovine serum and 1% penicillin/streptomycin (all from Thermo Fisher, Waltham, MA, USA), kept at 37°C in 5% CO_2	Hepatoblastoma	Primary tumor	12	male	unknown	fetal	unknown	unknown	unknown	unknown
HuH7		JCRB0403	CVCL_0336		Hepatocellular carcinoma	Primary tumor	684	male	unknown	unknown	unknown	unknown	unknown	unknown
HepG2	American Type Culture Collection, Manassas, VA	HB-8065	CVCL_0027		Transitional liver cell tumor	Primary tumor	180	male	unknown	unknown	unknown	unknown	unknown	unknown
Hep3B		HB-8064	CVCL_0326		Hepatocellular carcinoma	Primary tumor	96	male	unknown	unknown	unknown	unknown	unknown	unknown
HepT1	Dr. T. Pietsch (University of Bonn, Germany)	none	CVCL_G003		Hepatoblastoma	Primary tumor	34	female	IV	embryonal	No	Yes	No	DOD
PDX214	Established by XenTech (Evry, France)	none	none	Advanced DMEM/F12 containing 10 mg/L insulin supplemented with 10% fetal bovine serum, 1% penicillin/streptomycin, 1% L-glutamine (all from Thermo Fisher), as well as 20 μM Y-27632 (Selleckchem, Chesterbrook, PA), kept at 37°C in 5% CO_2	Hepatoblastoma	Primary tumor	31	female	II	fetal	Yes	Yes	Yes	RD
PDX243		none	none		Hepatoblastoma	Intrahepatic recurrence	52	male	unknown	embryonal	Yes	Yes	No	RD
PDX282		none	none		Hepatoblastoma	Primary tumor	12	male	II	embryonal	No	No	No	RD
PDX295		none	none		Hepatoblastoma	Primary tumor	26	female	II	fetal	Yes	Yes	Yes	unknown
PDX303		none	none		Hepatoblastoma	Primary tumor	69	female	II	fetal	No	Yes	No	unknown
PDX344		none	none		Transitional liver cell tumor	Lung metastasis	45	male	IV	unknown	Yes	Yes	Yes	RD
PDX346		none	none		Hepatoblastoma	Lung metastasis	18	female	unknown	embryonal	unknown	unknown	Yes	RD
HDFa	American Type Culture Collection, Manassas, VA	PCS-201-012	none	DMEM supplemented with 10% fetal bovine serum and 1% penicillin/streptomycin (all from Thermo Fisher, Waltham, MA, USA) kept at 37°C in 5% CO_2	Normal skin	Primary dermal fibroblasts	adult	unknown	unknown	unknown	unknown	unknown	unknown	unknown
HDFn		PCS-201-010	none		Normal foreskin	Primary dermal fibroblasts	neonatal	unknown	unknown	unknown	unknown	unknown	unknown	unknown
BEAS-2B		CRL-3588	CVCL_0168		Normal lung	Bronchial epithelial cells	unknown	unknown	unknown	unknown	unknown	unknown	unknown	unknown

Supplementary Table 2: compound list

Abbreviation	Full name	Supplier	Catalog no.
17AAG	Tanespimycin (17-AAG)	Selleckchem	S1141
5-FU	Fluorouracil	Selleckchem	S1209
ADAVO	Adavosertib (AZD1775)	Selleckchem	S1525
ALECT	Alectinib	Selleckchem	S2762
ALI	Alisertib	Selleckchem	S1133
APREP	Aprepitant	Selleckchem	S1189
AZD7762	AZD7762	Selleckchem	S1532
BELINO	Belinostat	Selleckchem	S1085
BI-2536	BI-2536	Selleckchem	S1109
BIX-tri	BIX-01294- trihydrochlorid hydrat	Sigma- Aldrich	B9311
BORTE	Bortezomib	Selleckchem	S1013
BRIGA	Brigatinib (AP26113)	Selleckchem	S8229
CABOZ	Cabozantinib	Selleckchem	S1119
CARBO	Carboplatin	Selleckchem	S1215
CARFI	Carfilzomib	Selleckchem	S2853
CERIT	Ceritinib	Selleckchem	S7083
CIS	Cisplatin	Selleckchem	S1166
CM272	CM272	Selleckchem	S8812
COBI	Cobimetinib	Selleckchem	S8041
CRIZO	Crizotinib	Selleckchem	S1068
DECITAB	Decitabine (NSC 127716)	Selleckchem	S1200
DICOUM	Dicoumarol	Selleckchem	S4299
DINA	Dinaciclib	Selleckchem	S2768
DOCE	Docetaxel (RP56976)	Selleckchem	S1148
DOX	Doxorubicin	Selleckchem	S1208
ENSAR	Ensartinib (X396)	Selleckchem	S2934
ETINO	Entinostat	Selleckchem	S1053
ETOP	Etoposide	Selleckchem	S1225
FIME	Fimepinostat	Selleckchem	S2759
FLAVO	Flavopiridol	Selleckchem	S1230
GANE	Ganetespib (STA-9090)	Selleckchem	S1159
GEMCI	Gemcitabine	Selleckchem	S1714
GILTER	Giltertinib (ATCL01EX13)	Selleckchem	S7754
IFOS	Ifosfamide	Selleckchem	S1302
INCYCL	Incyclinide (CMT-3, COL-3)	MedChemExpress	HY-13648
IRINO	Irinotecan HCl Trihydrate	Selleckchem	S2217
JQ1	(+)- JQ1	Selleckchem	S7110
Li71-2	LIN28 inhibitor LI71	MedChemExpress	HY-123905
LORLA	Lorlatinib (PF-06463922)	Selleckchem	S7536
MARIMA	Marimastat (BB-2516)	Selleckchem	S7156
MBZ	Mebendazole	Selleckchem	S4610
ML334	ML334	Millipore	5059870001
NICLO	Niclosamide	Selleckchem	S3030
NIFUROX	Nifuroxazide	Selleckchem	S4182
NSC207895	NSC207895 (XI-006)	Selleckchem	S2678
OLA	Olaparib	Selleckchem	S1060
OXALI	Oxaliplatin	Selleckchem	S1224
PACLI	Paclitaxel	Selleckchem	S1150
PALBO	Palbociclib	Selleckchem	S4482
PALIFOS	Palifosfamide	Selleckchem	S5840
PANO	Panobinostat	Selleckchem	S1030
PAT-1251	Lenumlostat (GB2064)	MedChemExpress	HY-107422
PRT4165	PRT4165	Millipore	203630
PTC-028	PTC-028	Selleckchem	S8662
PTC-209	PTC-209	Selleckchem	S7372
PTC209-HBr	PTC-209 Hbr	Selleckchem	S7539

PTC-596	PTC-596	Selleckchem	S8820
PXS-5153A	PXS-5153A monohydrochloride	MedChemExpress	HY-114286
QUISINO	Quisinostat	Selleckchem	S1096
RAPA	Rapamycin	Stemcell Tech	73364
ROMI	Romidepsin	Selleckchem	S3020
SN-38	SN-38	Selleckchem	S4908
SORA	Sorafenib	Selleckchem	S7397
SPAUT	Spautin-1	Sigma- Aldrich	SML0440
STYLO	Stylopine (Tetrahydrocoptisine)	Selleckchem	S3304
TAZEM	Tazemetostat (EPZ-6438)	Selleckchem	S7128
TRAME	Trametinib	Selleckchem	S2673
UNC3866	UNC3866	Selleckchem	S8359
VALPRO	Valproic acid sodium salt	Selleckchem	S1168
VCR	Vincristine	Selleckchem	S1241
VENETO	Venetoclax	Selleckchem	S8048
VER155008	VER155008	Selleckchem	S7751
VERTE	Verteporfin	Selleckchem	S1786
VINBLAST	Vinblastine sulfate	Selleckchem	S4505
VINOREL	Vinorelbine ditartrate	Selleckchem	S4269
VISMO	Vismodegib (GDC-0449)	Selleckchem	S1082
VOLA	Volasertib	Selleckchem	S2235
VORINO	Vorinostat (SAHA)	Selleckchem	S1047

Supplementary Table 3: antibody information

Abbreviation	Full name primary antibody	Supplier	Catalog no.	RRID	Host	Dilution	Full name secondary antibody	Supplier	Catalog no.	RRID	Dilution	application
Ac-H3	Acetyl Histone 3 (K14)	Cell Signaling	7627	AB_10839410	Rabbit	1:500	Anti-rabbit IgG, HRP-linked	Cell Signaling	7040S	AB_3391724	1:2000	Western blotting
AFP	Alfa-fetoprotein	Acris Antibodies	DM129	AB_972692	Mouse	1:500	Alexa Fluor 555 anti-mouse IgG	Invitrogen	A21422	AB_2535844	1:500	Immunofluorescence
DUSP1	Dual specificity phosphatase 1	Abcam	ab61201	AB_941356	Rabbit	1:1000	Anti-rabbit IgG, HRP-linked	Cell Signaling	7040S	AB_3391724	1:2000	Western blotting
EPCAM	Epithelial cell adhesion molecule [1B7]	Thermo fisher	14-9326-82	AB_795876	Mouse	1:250	Alexa Fluor 488 anti-mouse IgG	Invitrogen	A11001	AB_2534069	1:500	Immunofluorescence
ERK 1/2	Extracellular-signal regulated kinases 1 and 2	Cell Signaling	4695	AB_390779	Rabbit	1:1000	Anti-rabbit IgG, HRP-linked	Cell Signaling	7040S	AB_3391724	1:2000	Western blotting
GAPDH	Glycerinaldehyd-3-phosphat-dehydrogenase [6C5]	Thermo fisher	AM4300	AB_2536381	Mouse	1:10000	Anti-mouse, HRP-linked	Dako	P0447	AB_2617137	1:20000	Western blotting
GPC3	Glypican-3	Thermo fisher	PA5-77986	AB_2735861	Rabbit	1:250	Alexa Fluor 555 anti-rabbit IgG	Invitrogen	A27039	AB_2536100	1:500	Immunofluorescence
LMNB1	Lamin B1 [D9V6H]	Cell Signaling	13435S	AB_2737428	Rabbit	1:2000	Anti-rabbit IgG, HRP-linked	Cell Signaling	7040S	AB_3391724	1:4000	Western blotting
MYC	Myelocytomatosis oncogene	Cell Signaling	9402	AB_2151827	Rabbit	1:1000	Anti-rabbit IgG, HRP-linked	Cell Signaling	7040S	AB_3391724	1:2000	Western blotting
MYC	Myelocytomatosis oncogene	Cell Signaling	9402	AB_2151827	Rabbit	1:250	Alexa Fluor Plus 647 anti-rabbit IgG	Invitrogen	A32733	AB_2633282	1:500	Immunofluorescence
P-ERK 1/2	Phospho- ERK 1 and 2 (T202/Y204)	Cell Signaling	4370	AB_2315112	Rabbit	1:1000	Anti-rabbit IgG, HRP-linked	Cell Signaling	7040S	AB_3391724	1:2000	Western blotting
P-MYC	Phospho- MYC (S62) [EPR17924]	Abcam	ab185656	AB_2935659	Rabbit	1:1000	Anti-rabbit IgG, HRP-linked	Cell Signaling	7040S	AB_3391724	1:2000	Western blotting
SP-C	Surfactant protein C	Seven Hills	76694	AB_2938817	Rabbit	1:500	Alexa Fluor 488 anti-rabbit IgG	Invitrogen	A11008	AB_143165	1:500	Immunofluorescence
TUBA	Alfa-Tubulin [DM1A]	Sigma	T9026	AB_477593	Mouse	1:4000	Anti-mouse, HRP-linked	Dako	P0447	AB_2617137	1:10000	Western blotting
VIM	Vimentin [V9]	Santa-Cruz	sc-6260	AB_628437	Mouse	1:500	Alexa Fluor 647 anti-mouse IgG1 (γ)	Invitrogen	A21240	AB_2535809	1:500	Immunofluorescence
